# Magnetic Resonance Imaging Findings in 13 Neurologic Pot-Bellied Pigs

**DOI:** 10.3389/fvets.2020.00021

**Published:** 2020-01-31

**Authors:** Aude Castel, Vincent Doré, Mariana Vigeral, Silke Hecht

**Affiliations:** ^1^Department of Clinical Sciences, Faculty of Veterinary Medicine, University of Montreal, Saint-Hyacinthe, QC, Canada; ^2^Faculty of Veterinary Medicine, CHUV, Farm Animal Hospital, University of Montreal, Saint-Hyacinthe, QC, Canada; ^3^Department of Small Animal Clinical Sciences, College of Veterinary Medicine, University of Tennessee, Knoxville, Knoxville, TN, United States

**Keywords:** MRI, porcine, swine, neurology, brain, spine

## Abstract

This study reports the magnetic resonance imaging (MRI) findings in 13 pot-bellied pigs presented to our institution with neurological deficits. Nine pigs had abnormal MRI findings (7 with spinal cord localization and 2 with brain localization), with three of them having histopathological confirmation of the diagnoses. MRI diagnoses included a myopathy suspected to be secondary to *Erysipelothrix rhusiopathiae*, a round cell neoplasia involving the vertebral canal, myelomalacia, a cervical cyst like extradural lesion, pelvic fracture with secondary cauda equina involvement, two cases of fibrocartilaginous embolism or acute non-compressive nucleus pulposus extrusion, multifocal brain infarcts, and a cystic fourth ventricle dilation resulting in obstructive hydrocephalus and syringomyelia. Four pigs had normal MRI studies, with one of them ultimately diagnosed with idiopathic vestibular disease. This retrospective study illustrates the wide variety of diagnoses achieved with MRI of the head or vertebral column in pigs, several of them having never been described in this species. Some of the conditions identified had a good outcome. This justifies using MRI as an ante-mortem diagnostic tool as it can provide relevant information about the prognosis which can significantly influence treatment recommendations. Our findings suggest that MRI should be considered as a valuable imaging modality, when feasible, in pigs with neurological deficits.

## Introduction

Infectious diseases such as bacterial meningitis and myelitis, porcine teschoviruses, Porcine circovirus type 2, Porcine reproductive and respiratory syndrome, pseudorabies, metabolic and toxic causes such as hypoglycemia, salt and selenium toxicity or edema disease, and congenital abnormalities are the most common causes of neurological deficits in pigs (1). These diagnoses are generally achieved post-mortem because many pigs presented with acute neurologic conditions are ultimately culled as they are typically commercial pigs and ante-mortem diagnostics are usually not performed. Given the recent increase in popularity of pot-bellied pigs as pets, an interest in pursuing non-invasive, ante-mortem diagnostics is becoming more prevalent among owners. Magnetic Resonance Imaging (MRI) is the imaging modality of choice for most neurologic conditions in people and veterinary species. Certain MRI findings can provide key ante-mortem diagnostic information for veterinarians, allowing choice of appropriate treatment options in dogs and cats (2, 3). Similar information for the diagnosis of neurologic diseases in pigs is lacking, as only one case report has been published so far and the MRI study was performed post-mortem (4). The aim of this retrospective study was to describe the clinical presentation, MRI features, and outcome of neurologic conditions identified in a group of pot-bellied pigs, some with confirmed histolopathologic diagnosis.

## Materials and Methods

The MRI database of the Radiology service at the University of Tennessee Veterinary Medical Center was searched for any pig undergoing MRI between March 2008 and August 2019. Thirteen cases were identified. Case information retrieved included signalment, onset, progression, lateralization, and duration of signs when available, abnormalities on neurologic examination, head or vertebral column MRI findings, and follow up information including histopathological diagnosis when available. MRI was performed under general anesthesia with the animals placed in dorsal recumbency. Examinations were performed using either a 1T or a 1.5 Tesla unit (Siemens Harmony or Siemens Espree, respectively, Siemens Medical Solutions, Malvern, PA, USA). MRI studies were tailored to individual patients. Head MRI examinations typically included sagittal T2-weighted (T2W), transverse T2W, T1-weighted (T1W), T2-weighted fluid attenuation inversion recovery (T2-FLAIR), T2^*^-weighted gradient recalled echo (T2^*^GRE), and post gadolinium-based contrast T1W sequences in three planes. Vertebral column MRI examinations typically included dorsal short tau inversion recovery (STIR), sagittal T2W, T1W, STIR, and half-Fourier acquisition single-shot turbo spin-echo magnetic resonance imaging (“HASTE”), transverse T1W and T2W, and post contrast fat saturated sagittal and transverse T1W sequences. Contrast medium (Magnevist^®, ©^; Bayer HealthCare Pharmaceuticals Inc., Germany, USA) was administered intravenously at a dose of 0.1 mmol/kg.

## Results

Thirteen pigs were identified with our MRI database search. Four of these subjects had a normal MRI examination. The other nine had abnormalities noted on either head or vertebral column MRI. Further details on each case are provided in Supplementary Table 1. All pigs were confirmed to be pot-bellied pigs. Of these, nine animals were male (eight neutered and one intact) and four were female (three intact and one spayed), with an age range of 3 months−13 years (median, 12 months). The weight ranged from 7.2 to 145 kg with a median of 33 kg. Prior to presentation, the duration of neurologic signs ranged from 7 h to 1 month with a median of 7 days. Cerebrospinal fluid (CSF) analysis was performed in three pigs, and histopathological evaluation was performed in three animals.

### Cases With Myelopathy

#### Case 1

**Figure 1:**
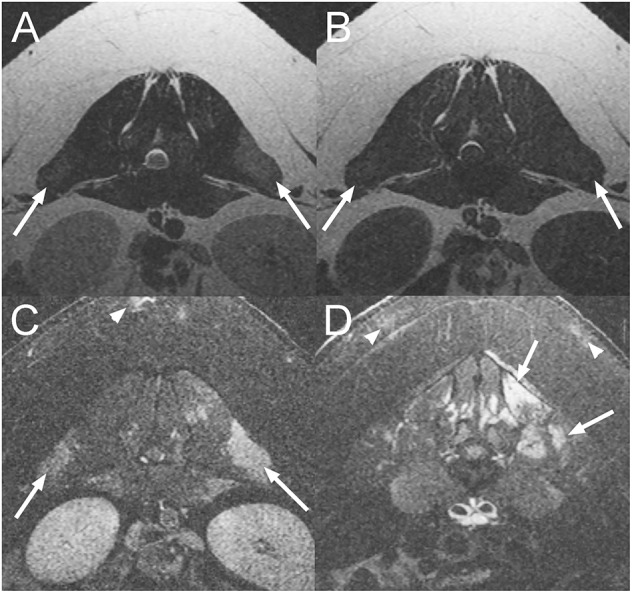
Transverse T2-weighted (T2W) **(A)**, T1-weighted (T1W) **(B)**, and post contrast T1W images with fat suppression **(C,D)** at the level of L1-2 intervertebral disc space **(A–C)** and L5 vertebra **(D)** of an 18-month-old pig diagnosed with a myopathy secondary to *Erysipelothrix rhusiopathiae* infection (Case 1). There are multifocal T2W hyperintense and T1W iso- to mildly hyperintense lesions when compared to normal muscle in the epaxial musculature of the lumbar vertebral column which are strongly contrast enhancing (arrows). Similar lesions are also associated with the subcutaneous fat (arrowheads).

An 18-month-old male castrated pig presented with a 10-day history of waxing and waning paraparesis, fever, and anorexia that had developed acutely. At presentation, the animal was febrile (105°F). Physical examination abnormalities included keratinized skin lesions characterized by crusting on the neck and the lumbar area, circular lesions of the ventrum, as well as bilateral pelvic limb paresis. A L(lumbar)4–S (sacral)3 myelopathy was suspected but the complete neurological examination was not documented. Differential diagnoses included a bacterial meningomyelitis of hematogenous origin, an epidural abscess, severe bacterial dermatitis (*Erysipelothix rhusiopathiae, Staphylococcus hyicus* or *Staphylococcus aureus*) causing secondary myositis, osteomyelitis and/or disconspondylitis or less likely a trauma to the spinal cord. On MRI of the lumbosacral vertebral column (Figure 1), there are multifocal T2W hyperintense and T1W iso- to mildly hyperintense to normal muscle lesions in the lumbar epaxial musculature. Following contrast medium administration, there is intense, patchy, ill-defined enhancement of these lesions and of the dorsal subcutaneous fat of the lumbar vertebral column. These findings are consistent with myositis and steatitis. Biopsies of the lumbar epaxial muscles and of the skin were collected and fixed in formalin. Histopathology of the skeletal muscles was consistent with myofiber necrosis and degeneration with lymphohistiocytic inflammation and regeneration. Histopathology from the skin lesions showed marked epidermal hyperplasia with hyperkeratosis, moderate lymphoplasmacytic and eosinophilic perivascular dermatitis, and multifocal follicular atrophy. Some sections revealed bacterial cocci within the surface crust. Immunohistochemistry of these skin lesions was positive for *Erysipelothrix rhusiopathiae*. The pig improved after 5 days of treatment with cephalexin (Keflex^©^; Advancis Pharmaceutical Corp., USA; 22 mg/kg; BID; PO) and carprofen (Rimadyl^©^; Zoetis, USA; 2.2 mg/kg; BID; PO).

#### Case 2

**Figure 2:**
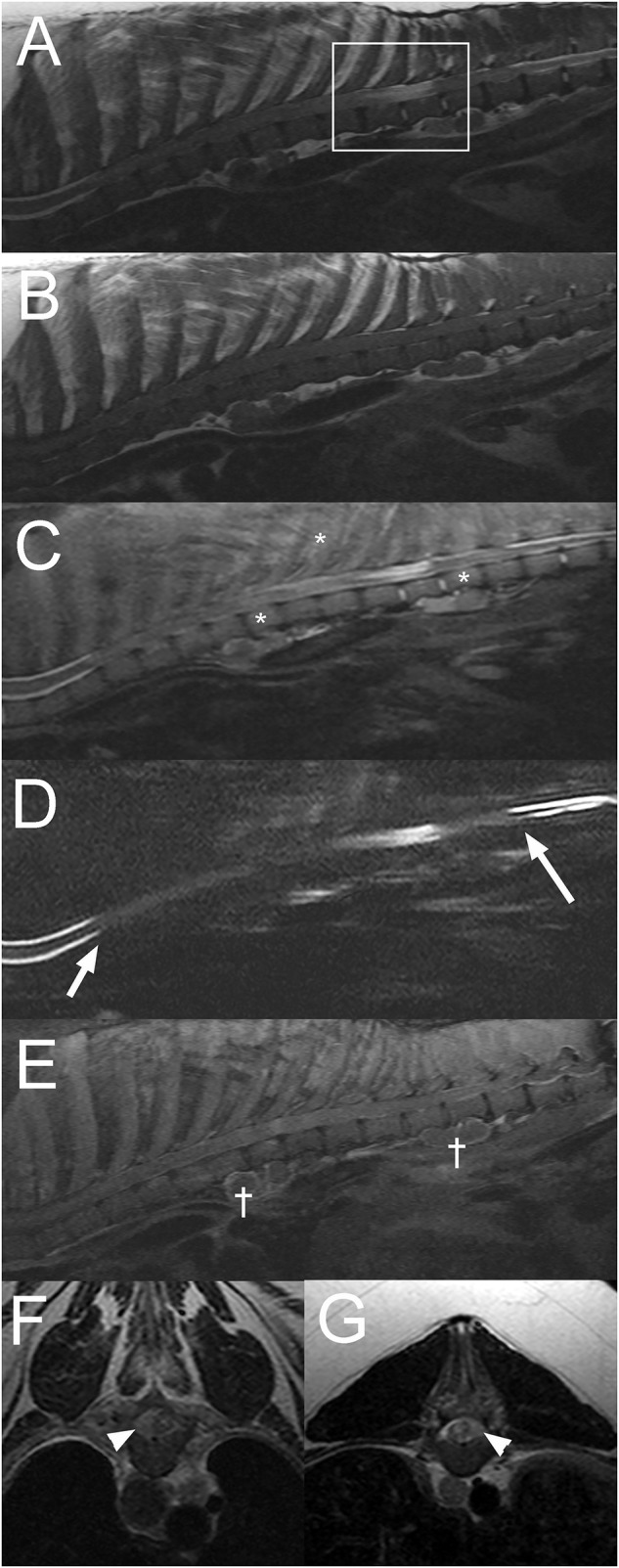
Sagittal T2W **(A)**, T1W **(B)**, short tau inversion recovery (STIR) **(C)**, half-Fourier acquisition single-shot turbo spin-echo magnetic resonance imaging (HASTE) **(D)**, post contrast T1W fat saturated images **(E)**, and transverse T2W images at the level of T6-7 **(F)**, and T11-12 **(G)** intervertebral disc spaces of the thoracolumbar vertebral column in a 13-year-old pig with a final diagnosis of diffuse round cell neoplasia (Case 2). Note the extensive attenuation of the subarachnoid space and epidural fat from the T3 to T13 vertebrae (**A–D**; arrows in “**D**”), intramedullary lesion at T10-11 vertebrae (box in “**A**”), faint patchy multifocal STIR hyperintensity of vertebral bodies and paraspinal soft tissues (**C**; *). There is multifocal contrast enhancement of extradural material, meninges, vertebrae, paraspinal soft tissues, and para-aortic lymph nodes (^†^ in “E”). On transverse images, there is evidence of extradural spinal cord compression [right sided at the T6-7 intervertebral disc space **(F)** and left sided at the T11-12 intervertebral disc space **(G)**] (arrowheads).

A 13-year-old male castrated pot-bellied pig presented with a 4-week history of paraplegia. The animal had become acutely unable to use the pelvic limbs and had been unchanged since. On presentation, the pig was paraplegic with increased muscle tone in the pelvic limbs, intact reflexes, and a Schiff-Sherrington posture suggesting a T(thoracic)3-L3 myelopathy. Mentation and cranial nerve examination was normal. Presence of superficial and deep nociception, as well as reaction to vertebral column palpation were not reported. Differential diagnoses included an ischemic myelopathy such as fibrocartilaginous embolism (FCE), neoplasia involving the vertebral canal or the spinal cord and degenerative changes to the vertebrae causing secondary spinal cord compression. A traumatic injury could not be completely excluded but was considered less likely. On MRI of the thoracic vertebral column (Figure 2), there is extensive extradural material causing attenuation of the normal hyperintense signal from CSF and epidural fat from T3 to T13 vertebrae on sagittal T2W images. An intramedullary T2W and STIR hyperintensity (compared to normal spinal cord parenchyma) is present at the level of T10-11 vertebrae. On the transverse images, there is marked right-sided spinal cord compression at the level of T6-7 vertebrae and moderate left sided spinal cord compression at T11-12 vertebrae due to extradural material isointense on T1W and slightly hyperintense to the spinal cord on T2W images. Following contrast medium administration, there is patchy, ill-defined enhancement of the extradural material as well as mild multifocal meningeal and very mild vertebral enhancement. Ventral to the thoracic and lumbar vertebral column, there are numerous T2 and STIR hyperintense (to the bone), round to ovoid, para-aortic well-defined structures, surrounded by STIR hyperintense tissue (compared to bone) and showing rim enhancement following contrast medium administration. These lesions are consistent with enlarged para-aortic lymph nodes. These findings raised the concern for a disseminated neoplastic process, and given the poor prognosis, the pig was euthanized and submitted for necropsy.

The histopathology results were consistent with diffuse round cell neoplasia infiltrating the spinal cord, vertebrae, liver, spleen, lung, and lymph nodes. Definitive characterization of the type of neoplasia was not possible as although the immunohistochemistry was negative for B-cell (CD79), T-cell (CD3), and plasma cell (Mum-1), the canine markers used have not been studied on pig tissue.

#### Case 3

**Figure 3:**
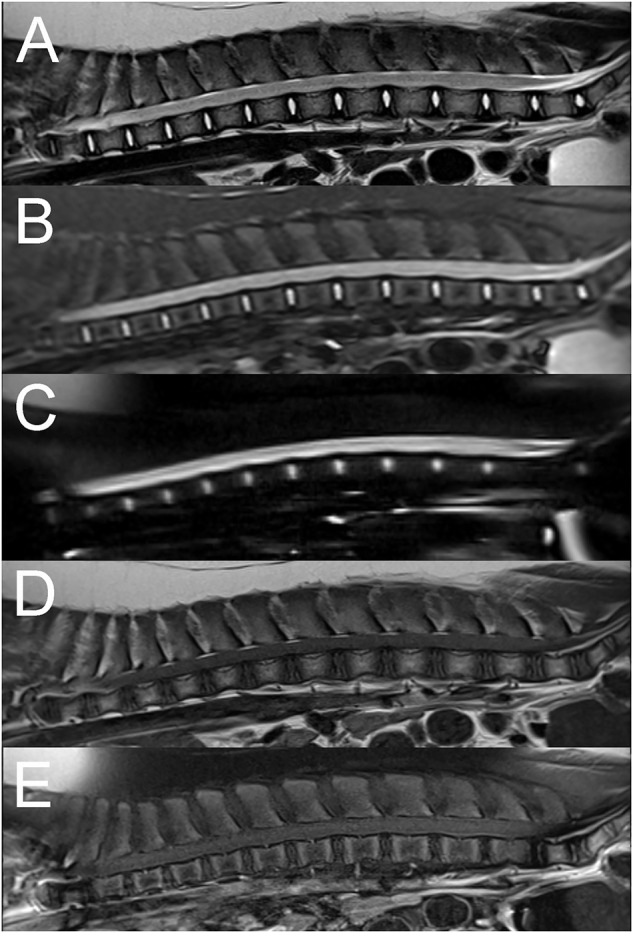
Sagittal T2W **(A)**, STIR **(B)**, HASTE **(C)**, pre **(D)**, and post contrast T1W fat saturated **(E)** images of the thoracolumbar vertebral column in a 6-month old pig with complete sensorimotor loss 4 days after jumping off the bed (Case 3). There is extensive diffuse intramedullary T2W and STIR hyperintensity compared to normal spinal cord parenchyma and spinal cord swelling associated with circumferential attenuation of the subarachnoid space throughout the entire length of the thoracolumbar vertebral column **(A–C)**. The intramedullary lesion is isointense to the spinal cord on T1W images **(D)** with no evidence of abnormal contrast enhancement **(E)**. The findings were suggestive of myelomalacia, likely secondary to a spinal cord contusive injury.

A 6-month-old male castrated pig presented acutely paraplegic after jumping from the bed 4 days prior to presentation. Neurological examination findings revealed paraplegia without deep nociception and intact spinal reflexes. The rest of the examination was normal. Presence of pain on vertebral column palpation was not reported. A T3-L3 myelopathy was suspected based on neurological examination findings. Differential diagnoses included a vertebral fracture or luxation with subsequent spinal cord injury, an intervertebral disc extrusion or an acute non-compressive nucleus pulposus extrusion (ANNPE). Computed tomography (CT) of the vertebral column was unremarkable. MRI shows extensive intramedullary hyperintensity to the spinal cord throughout the entire thoracolumbar spinal cord on T2W and STIR sequences (Figure 3). These changes are isointense to the spinal cord on T1W images. Additionally, there is spinal cord swelling and attenuation of the subarachnoid signal on T2W, STIR, and HASTE sequences. These findings were consistent with extensive myelomalacia and, since there was no evidence of a compressive extradural lesion, a contusive injury to the spinal cord was considered most likely. Given the severity of the neurological deficits, the animal was euthanized. The owner did not consent to a post-mortem evaluation.

#### Case 4

**Figure 4:**
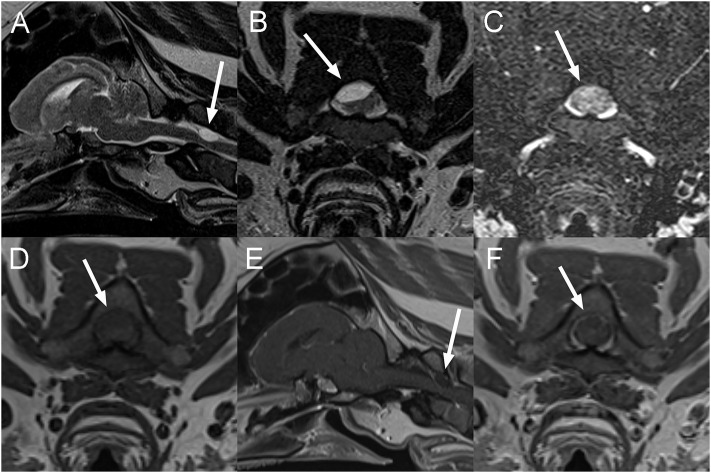
Sagittal T2W **(A)**, transverse T2W **(B)**, T2-Fluid attenuation inversion recovery (FLAIR) **(C)**, T1W **(D)**, and sagittal **(E)**, and transverse **(F)** post contrast T1W images of the head and cranial cervical vertebral column in a 10-year-old pig presented with progressive deficits attributable to a brainstem or cervical spinal cord lesion (Case 4). There is a T2W hyperintense well-circumscribed ovoid mass lesion associated with the right dorsal aspect of the vertebral canal, resulting in marked displacement and compression of the spinal cord from right dorsal **(A,B)**. The lesion remains mildly hyperintense on T2-FLAIR **(C)** and is hypointense to the spinal cord on T1W images **(D)** with very faint linear peripheral contrast enhancement adjacent to the lesion **(E,F)**. The lesion is indicated by arrows on all images. An extradural cyst-like lesion containing cellular or proteinaceous fluid was considered most likely.

A 10-year-old male castrated pot-bellied pig presented for a 1-week history of progressive ataxia and right thoracic limb monoparesis. The owner was out-of-town, noted the deficits upon return and did not know about the onset of signs. On presentation, the pig was quiet and responsive with right thoracic limb paresis. A right-sided head tilt had been noted the day before by the owner but was absent at the time of examination. At that time, it was unclear whether the pig was uncomfortable. The rest of the neurological examination was normal. Over the course of 1 day, the pig worsened and became severely ataxic (proprioceptive), dull, and tetraparetic worst on the right. Spinal reflexes and cranial nerves examination were intact. The animal progressed to become non-ambulatory and it was unclear whether the pig's altered mentation was secondary to cervical pain or ascending reticular activated system involvement. Neuroanatomical localization was to the brainstem or the cervical spinal cord (C1 (cervical)-C5 myelopathy). Differential diagnosis included rabies, other causes of infectious meningoencephalomyelitis such as pseudorabies, *Streptococcus suis* and *Listeria monocytogenes* or a neoplastic process. A CBC and chemistry panel were within normal limits. MRI of the head and cranial cervical vertebral column (Figure 4) reveals a well-defined, ovoid, extradural mass (~1.3 × 0.7 cm), T2W hyperintense, and T1W hypointense to the spinal cord in the right dorsal aspect of the vertebral canal at the level of the cranial aspect of the body of C2, resulting in marked spinal cord compression. The spinal cord is deviated ventrally and toward the left. This mass remains hyperintense to the spinal cord on T2-FLAIR sequence and does not suppress on the STIR sequence. There is no evidence of susceptibility artifacts within the lesion on T2^*^GRE imaging. The center of the mass does not display contrast enhancement, but there is very mild peripheral enhancement. Based on these findings, an extradural cyst- like lesion containing proteinaceous fluid was considered most likely, but a granuloma or a neoplastic process could not be completely excluded. The patient was euthanized the same day and necropsy was declined.

#### Case 5

**Figure 5:**
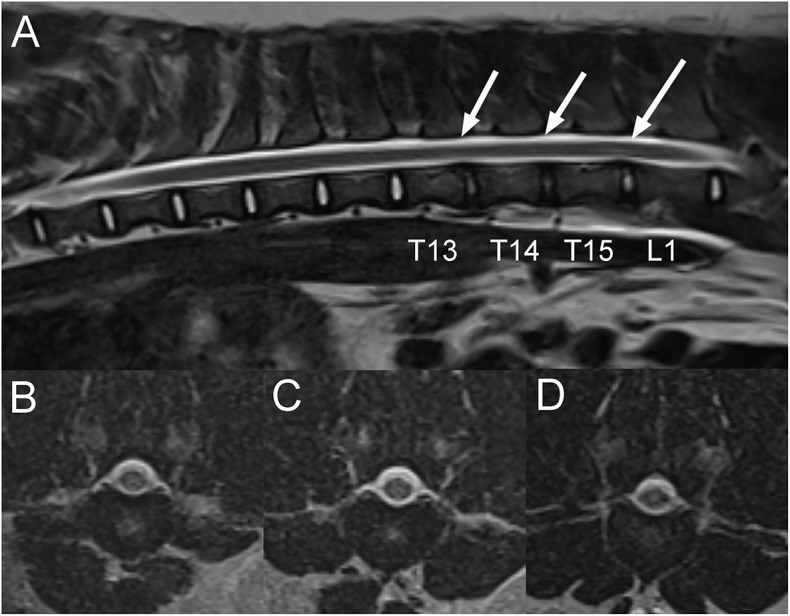
Sagittal T2W **(A)** and transverse T2W images of the thoracolumbar vertebral column at the level of T13-14 **(B)**, T14-15 **(C)** and T15-L1 intervertebral disc spaces in a 1-year-old pig with acute T3-L3 myelopathy (Case 5). There is mild decrease in normal T2W signal intensity of the T13-14, T14-15 and T15-L1 intervertebral discs compared to the other ones and very mild protrusion of the T15-L1 and T13-14 intervertebral discs. There are faint linear intramedullary T2W hyperintense areas compared to the spinal cord over the caudal thoracic and cranial lumbar vertebral column, mostly centered over the T15-L1 intervertebral disc space, less obvious over the T13-14 and T14-15 intervertebral disc spaces (**A**; arrows), and located within the central to right lateral aspect of the spinal cord on transverse images **(B–D)**. Based on clinical presentation and imaging findings, a presumptive diagnosis of either fibrocartilaginous embolism (FCE) or acute non-compressive nucleus pulposus extrusion (ANNPE) was made.

A 1-year-old intact female pot-bellied pig presented 1-month after becoming acutely unable to stand on the pelvic limbs. The animal had received an injection of corticosteroids and had shown some improvement since the initial onset of signs according to the owner. On neurologic examination, the pig was non-ambulatory paraparetic with intact spinal reflexes (withdrawal and patellar). Cranial nerve examination was unremarkable. No pain was noted on vertebral column palpation. A T3-L3 myelopathy was suspected with the differential diagnoses including an ischemic myelopathy such as a FCE, ANNPE, an intervertebral disc extrusion or less likely a vertebral fracture or luxation following an unwitnessed trauma. On MRI of the thoracolumbar vertebral column, there is mildly decreased normal T2W signal intensity of the T13-14, T14-15, and T15-L1 intervertebral discs compared to other discs, mild protrusion of the T15-L1 disc, and minimal protrusion of the T13-14 disc. Faint T2W intramedullary hyperintense (to the spinal cord) areas are centered mostly over the T15-L1 intervertebral disc space and minimally over the T13-14 and T14-15 disc spaces (Figure 5). Differential diagnoses in this patient based on imaging findings included FCE and ANNPE. The pig was discharged 1 day following admission showing improvement and was lost to follow up.

#### Case 6

**Figure 6:**
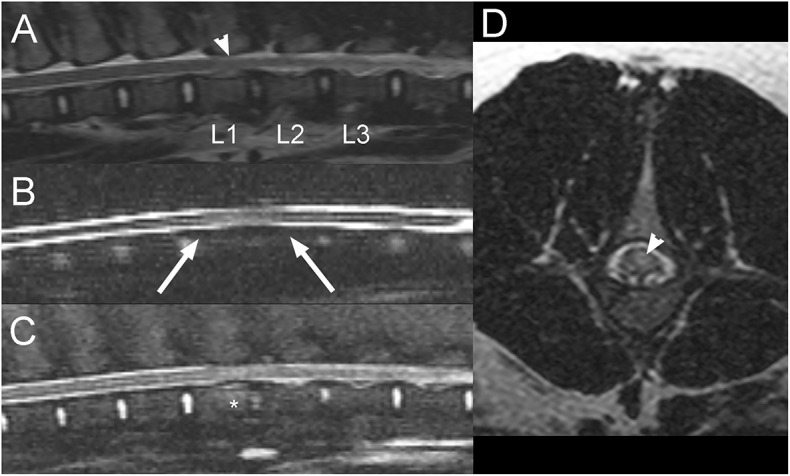
Sagittal T2W **(A)**, HASTE **(B)**, STIR **(C)**, and transverse T2W **(D)** images of the lumbar vertebral column in a 9-month-old pig with acute L4-S3 myelopathy (Case 6). There is decreased size and T2W signal intensity of the nucleus pulposus of the L1-L2 and to a lesser degree the L2-L3 intervertebral discs **(A)**. Partial attenuation of the dorsal and ventral CSF signal is noted on sagittal HASTE images with mild dorsal deviation of the ventral CSF column over L1-L2 intervertebral disc space (**B**; arrows). A small amount of extradural material is associated with the ventral aspect of the vertebral canal, but there is no evidence of significant spinal cord compression **(A,C)**. On STIR images, the caudal half of the L1 vertebral body is mildly hyperintense to the bone, consistent with edema or a bone contusion (**C**; *). There is mild diffuse T2 hyperintensity within the spinal cord at the level of L1-2 disc space, slightly more severe left sided (**A,D**; arrowheads). These findings were considered most consistent with ANNPE.

A 9-month-old, intact female pig presented after an acute onset of inability to stand on the pelvic limbs that had occurred the day of presentation. The owner reported vocalization at the time of onset, found the animal recumbent and suspected that the pig had been kicked by a horse. Neurological examination revealed paraplegia with intact superficial and deep nociception in the pelvic limbs. Patellar reflexes were decreased bilaterally. Withdrawal reflexes and anal tone were intact. Vertebral column hyperesthesia was not appreciated. Thoracic limbs and cranial nerve evaluation were unremarkable. A L4-S3 myelopathy and more precisely a lesion of the cranial aspect of the lumbar intumescence was suspected. Differential diagnoses included FCE, ANNPE, a vertebral fracture or luxation, or an intervertebral disc extrusion, although the latter were considered less likely given the lack of discomfort. On MRI of the lumbosacral vertebral column, there is decreased size and T2W signal intensity of the nucleus pulposus of the L1-L2 and to a lesser degree, the L2-L3 intervertebral discs compared to the other ones. There is also partial attenuation of the dorsal and ventral CSF signal on sagittal HASTE images with mild dorsal deviation of the ventral CSF column over L1-L2 intervertebral disc (Figure 6). A small amount of extradural material is associated with the ventral aspect of the vertebral canal with no evidence of significant spinal cord compression. On STIR images, the caudal half of the L1 vertebral body is mildly hyperintense to the bone, consistent with edema or a bone contusion. There is mild diffuse intraparenchymal T2W hyperintensity to the spinal cord at the level of L1-2 intervertebral disc, slightly more severe left sided. These findings were considered more consistent with ANNPE, rather than an ischemic myelopathy such as FCE. This pig underwent underwater treadmill sessions and hyperbaric oxygen chamber treatments while hospitalized. The animal showed significant improvement in neurologic status and was discharged 11 days after admission being able to stand up without assistance. Two years later, the pig appeared to be doing well with only mild residual ataxia.

#### Case 7

**Figure 7:**
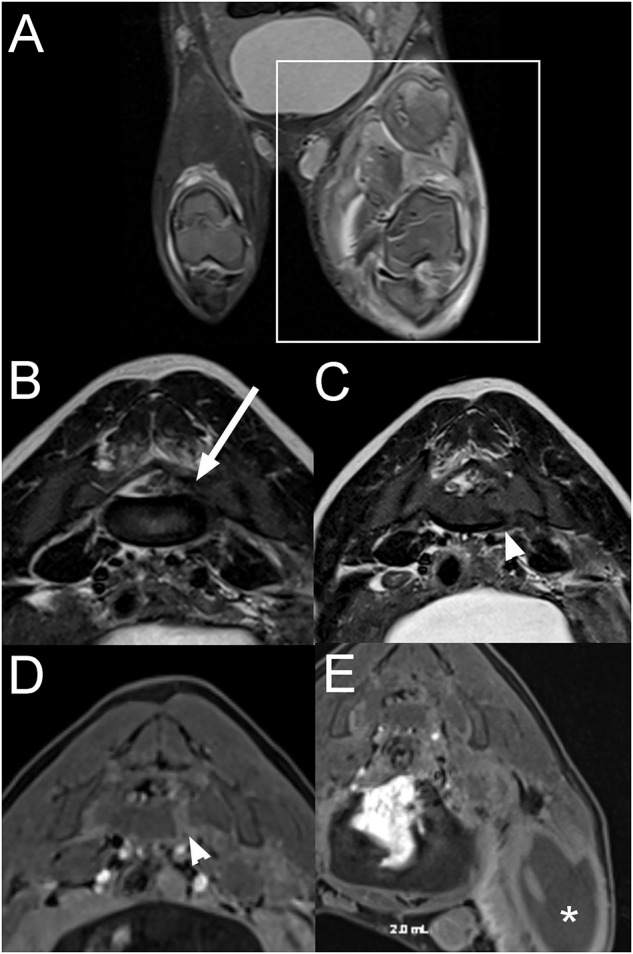
Dorsal STIR **(A)**, transverse T2W **(B,C)**, and transverse T1W post contrast fat suppressed **(D,E)** images of the pelvis and lumbosacral vertebral column in a 3-month-old pig with a L4-S3 myelopathy (Case 7). There is severe extensive swelling and STIR hyperintensity of the musculature of the left pelvic limb compared to normal muscle (**A**; within box) with large non-contrast enhancing areas surrounded by rim of contrast-enhancement (**E**; *). T2W hypointense to the spinal cord extradural material is present within the left side of the vertebral canal at the L6-S1 intervertebral disc space, extending into the left S1-S2 intervertebral foramen (**B**; arrow). There is a left parasagittal T2W hyperintense, T1W hypo- to hyperintense (to bone) and moderately contrast enhancing line associated with the body of the S1 vertebra with a step defect of the ventral cortex (**C,D**; arrowhead). The abnormalities were consistent with a fracture of the S1 vertebra with associated epidural hemorrhage, nerve root laceration, and left pelvic limbs muscle necrosis or abscessation.

A 3-month-old female intact pig presented after being found recumbent on the side of the road. On presentation, the pig was dull and unable to stand. A wound previously closed with staples was present on the right pelvic limb, and the left pelvic limb appeared diffusely swollen. On neurological examination, the pig was non-ambulatory with moderate paresis, delayed proprioceptive placement, decreased patellar, and normal withdrawal reflexes in the right pelvic limb. There was complete monoplegia with absent patellar and withdrawal reflexes and absent superficial and deep nociception on the left pelvic limb. Lumbosacral pain was also noted on palpation. The rest of the neurological examination was unremarkable. The neurolocalisation was L4-S3 myelopathy. Differential diagnoses included a vertebral fracture or luxation with subsequent spinal cord or nerve injury, an epidural abscess, myositis involving the left pelvic limb, cellulitis, or an abscess in the proximal portion of the pelvic limb with secondary constrictive neuropathy and less likely rabies. Blood work was consistent with an inflammatory leukogram, muscle damage, and thrombocytopenia. Radiographs of the left pelvic limb revealed gas within the soft tissues surrounding the left femur, consistent with a penetrating wound but no evidence of a fracture. Thoracic radiographs were within normal limits. On MRI of the pelvis (Figure 7), there is a moderate amount of extradural material T2W and T1W hypointense to the spinal cord within the left side of the vertebral canal at the L6-S1 intervertebral disc space, extending caudally along S1 vertebrae and into the left S1-S2 intervertebral foramen. The nucleus pulposus of the lumbosacral intervertebral disc is smaller. Associated with the body of S1 vertebrae, there is a left parasagittal T2W hyperintense, T1W hypointense to hyperintense (to the bone) and moderately contrast enhancing line, and the ventral cortex of S1 vertebrae has a step defect. Most muscles of the left thigh, left iliopsoas, and caudal left epaxial muscles are swollen, heterogeneously T2W hyperintense and surrounded by fluid. On post-contrast images, the imaged muscle of the left thigh and the left iliopsoas muscle display large non-contrast enhancing areas surrounded by a rim of mild to moderate contrast-enhancement and mild contrast-enhancement of the surrounding muscles. The left inguinal lymph node is markedly enlarged with a rim of moderate contrast-enhancement. The spinal abnormalities were consistent with a fracture of S1 vertebra with associated epidural hemorrhage, lacerated sacral nerve roots, or a combination of both. The changes to the left pelvic limb, iliopsoas, and caudal left epaxial muscles were consistent with trauma, myositis and areas of muscle necrosis or abscessation. The regional lymphadenopathy was considered most likely reactive.

The left pelvic limb was amputated at the level of the coxofemoral joint. Histopathologic examination yielded a final diagnosis of chronic, severe suppurative rhabdomyositis with bacterial cocci and fibrosis. The pig appeared more comfortable after the surgery but was still unable to walk. Three weeks later, the animal was improved significantly and was able to walk on three legs without deficits.

### Cases With a Neurolocalization to the Brain

#### Case 8

A 7-year-old male castrated pig presented for a 2-week history of incoordination and altered mentation. On physical examination, the pig was dull and had a proprioceptive ataxia in all four limbs. The rest of the neurological examination was not documented. Neuroanatomical localization was suspected to be to the forebrain or brainstem. Differential diagnoses included salt toxicity, thiamine deficiency, neoplasia, an infectious meningoencephalitis such as pseudorabies, Teschen disease, and less likely due to the age of the animal*, Streptococcus suis* and *Histophilus parasuis*. A complete blood count (CBC) revealed moderate anemia (PCV: 12.1%, ref.: 22–50; red blood cells count: 2.14 × 10^6^ cells/μL, ref.: 5.30–9.25) and a chemistry panel revealed findings consistent with azotemia (BUN: 102 mg/dL, ref.: 4.2–15.1; Creatinine: 9.9 mg/dL, ref.:.5–2.0), hyperglycemia (136 mg/dL, ref.: 56–123), hypoproteinemia (5.6 g/dL, ref.: 6.6–8.9) and hypoalbuminemia (1.4 d/dL, ref.: 2.9–5.6). A urinalysis revealed marked proteinuria and hematuria.

**Figure 8:**
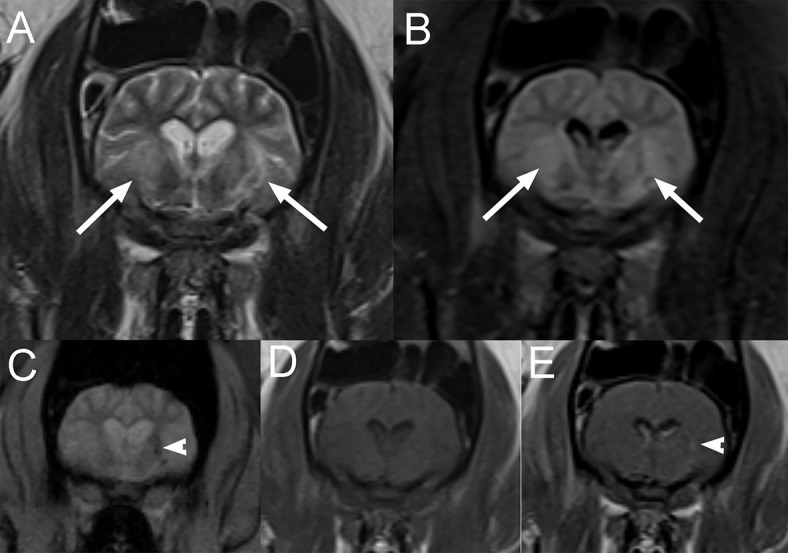
Transverse T2W **(A)**, T2-FLAIR **(B)**, T2*-weighted gradient recovery echo (T2*GRE) **(C)**, pre **(D)**, and post contrast **(E)** T1W images in a 7-year-old pig presented for progressive ataxia and mentation change and ultimately diagnosed with moderate multifocal acute and chronic encephalomalacia with thrombi (infarcts) secondary to endocarditis (Case 8). Note the poorly marginated heterogeneous T2W and T2-FLAIR **(A,B)** hyperintense lesions compared to white matter in the thalamus (arrows). Several of the lesions display susceptibility artifacts on T2*-GRE (**C**; arrowhead), consistent with hemorrhage and/or intravascular thrombi. These lesions are isointense to slightly hypointense to the white matter on T1W images **(D)** with faint and progressive enhancement seen within the left thalamic lesions (**E**; arrowhead).

On MRI of the brain (Figure 8), there are poorly marginated heterogeneous T2W and FLAIR hyperintense, and T1W iso- to slightly hypointense lesions when compared to normal brain in the thalamus and in the left piriform lobe. Several of the lesions display susceptibility artifacts on T2^*^GRE images, consistent with hemorrhage and/or intravascular thrombi. Following contrast medium administration, there is faint and progressive enhancement seen within the left thalamic lesions. Medical treatment for presumptive glomerulonephritis and thiamine supplementation were initiated, but due to the lack of response to treatment, the pig was euthanized and submitted for necropsy. Histopathologic findings were consistent with moderate multifocal acute and chronic encephalomalacia with thrombi (infarcts), marked diffuse necrotizing glomerulonephritis with protein casts and interstitial fibrosis, marked multifocal acute and chronic myocardial necrosis, and marked acute fibrinosuppurative endocarditis with intralesional bacteria.

#### Case 9

**Figure 9:**
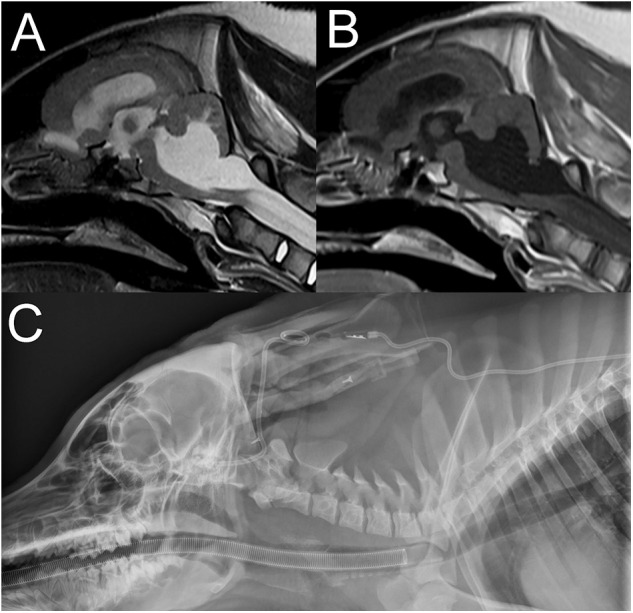
Sagittal T2W **(A)** and T1W **(B)** images of the head and cranial cervical region in a 10-month-old pig presented for progressive vestibular signs (Case 9). There is severe cyst-like dilation of the 4th ventricle and cerebellomedullary cistern causing marked dorsal deviation and compression of the cerebellum and ventral deviation with compression of the brain stem and cranial cervical spinal cord. There is also moderate to severe dilation of the olfactory recesses and lateral and 3rd ventricles. The cervical spinal cord is diffusely hyperintense to normal spinal cord on T2W **(A)** and hypointense on T1W **(B)** images. These findings are consistent with a congenital dilation of the 4th ventricle or a 4th ventricular diverticulum with secondary obstructive hydrocephalus and syringohydromyelia. Lateral radiograph of the head and neck following surgical ventriculoperitoneal shunt placement **(C)**. Thoracic and abdominal radiographs (not shown) confirmed appropriate tube location and termination within the peritoneal cavity.

A 10-month-old male castrated pig presented for progressive neurological deficits including disorientation, ataxia, circling, and pathologic nystagmus. The pig had been treated for 2 weeks with meloxicam for suspected neck pain but had developed new neurological signs since. On presentation, the animal was dull and circling to the right side. Vestibular ataxia and proprioceptive deficits to the left were noted. A vertical nystagmus was also present. The rest of the neurological examination was unremarkable. Based on the deficits, the neurolocalization was to the vestibular system and more precisely to the brainstem to explain the dullness and proprioceptive deficits with possibly left cerebellar involvement (including the caudal peduncle or flocculonodular lobe) to explain the paradoxical vestibular signs with the vestibular ataxia and circling to the right. Differential diagnoses included otitis media interna with intracranial extension, a meningoencephalitis from a viral source (pseudorabies, Teschen disease, and rabies), or vector-borne diseases (ehrlichia, rickettsia). On MRI of the head, there is severe cyst-like dilation of the 4th ventricle and cerebellomedullary cistern causing marked dorsal deviation and compression of the cerebellum and ventral deviation with compression of the brainstem and cranial cervical spinal cord (Figure 9). There is also moderate to severe dilation of the olfactory recesses and lateral and 3rd ventricles. The cervical spinal cord is diffusely hyperintense on T2W and hypointense on T1W images. The cerebellar vermis immediately dorsal to the 4th ventricle is T2W and FLAIR hyperintense and mildly T1W hypointense, consistent with edema. These findings were consistent with a congenital dilation of the 4th ventricle or a 4th ventricular diverticulum with secondary obstructive hydrocephalus and syringohydromyelia. A lumbar CSF collection and analysis showed a mild, mixed, predominantly mononuclear pleocytosis (total protein: 18.6 mg/dL, red blood cell count: 1,642 cells/μL, total nucleated cell count: 31 cells/μL). The pig was taken to surgery and a ventriculoperitoneal shunt was placed into the 4th ventricle. The pig recovered from the surgery and on discharge 5 days later, a right-sided head tilt, vestibular ataxia and vertical nystagmus were the only abnormalities on the neurological examination. The circling and proprioceptive deficits had resolved. Upon recheck examination 2 weeks later, the pig was improved neurologically, showing only mild residual vestibular ataxia and as well as a right-sided head tilt. These deficits persisted at the 4-week recheck examination. Unfortunately, 6 weeks following surgery, the pig became febrile and worsened neurologically. Infection of the shunt and secondary septic meningitis were suspected based on CSF analysis showing a neutrophilic pleocytosis (TNCC: 173/μL, RBC: 398/μL and TP: 88.6 mg/dL, 61% neutrophils, 31% lymphocytes, and 8% macrophages). Despite broad-spectrum antibiotics, the pig failed to improve and was euthanized. Necropsy was declined.

### Cases With Normal MRI Examinations

Four pigs presented for neurologic deficits, and no abnormalities were seen on MRI examination. Two patients presented with acute vestibular signs. One animal with vestibular ataxia, and a pathologic horizontal nystagmus (fast phase to the right) was diagnosed with meningoencephalitis based on an inflammatory lumbar CSF analysis (TP: 27 mg/dL; TTNC: 40/μL; RBC:1,990/μL) and was lost to follow up. The second animal was diagnosed with presumptive idiopathic vestibular syndrome based on a normal CSF analysis and negative infectious disease tests. On presentation, the neurological examination of this pig could not differentiate between lesions in the central or in the peripheral vestibular system as the severity of the pig's signs (restlessness, rolling, and thrashing) required the animal to be sedated for any manipulation. This pig slowly improved neurologically over the next few months. Another pig presented for a suspected seizure, had a normal brain MRI examination, and remained seizure-free for the next 3 years following the event. The last pig, a 3-month-old male, presented for reluctance to put weight on its pelvic limbs. Onset and progression of signs were unclear as the animal had been surrendered to a rescue organization. Neurological examination was abbreviated due to the pig's temperament but revealed weakly ambulatory paraparesis with a short strided gait, delayed hopping, and proprioceptive placement. The withdrawal reflexes appeared diminished and the patellar reflexes could not be reliably assessed, as the pig would not tolerate restraint. Cranial nerve examination appeared unremarkable although the menace response was inconsistent which was attributed to the animal's temperament. The presence of pain on spinal palpation was difficult to assess as the pig was vocalizing with every manipulation. A L4-S3 myelopathy was suspected and the differential diagnoses included a trauma (spinal cord contusion, vertebral fracture/luxation), congenital malformation, *Streptococcus suis* or *Haemophilus parasuis* meningomyelitis and much less likely metabolic disturbances (salt toxicity and hypoglycemia). MRI of the lumbosacral vertebral column was normal and the pig improved with a combination of gabapentin, methocarbamol, and anti-inflammatory corticosteroids.

## Discussion

This study describes MRI features of different etiologies causing neurological deficits in pigs. Several of these conditions have not yet been reported in swine and should potentially be added as differential diagnoses when neurological deficits are present in this species. Although some of these diseases such as FCE, ANNPE, vascular events, and idiopathic vestibular disease are common in dogs and cats and frequently diagnosed by neurologists, large animal veterinarians might not be as familiar with them. Knowing that these differential diagnoses should be considered in pigs could change treatment recommendations and the outcome for these animals. Furthermore, MRI can provide relevant information about the prognosis for a particular animal and either justify treatment when there is a chance of improvement or help with euthanasia decision when the outcome appears poor.

In one pig, the MRI findings were consistent with an extradural neoplasm compressing the spinal cord and with vertebral involvement. Based on histopathological findings, a round cell neoplasm was suspected. Extradural tumors are reported in ~50% of cases of spinal cord tumors in dogs, whereas intradural-extramedullary tumors represent 35% and intramedullary tumors make up the last 15% (5–8). Lymphoma has commonly been associated with involvement of more than one spinal compartment, including vertebrae and vertebral canal, and causing spinal cord compression (9). Unfortunately, in this case, further determination of the type of round cell neoplasm could not be achieved. Neoplasms reported to cause neurological signs in pigs include osteogenic sarcoma, melanoma, multiple myeloma, and glioblastoma (1). However, these diagnoses were only achieved with post-mortem evaluation and therefore, we are detailing the first report of MRI features of a spinal neoplasm in a pig. With increased popularity in pet pig, veterinarians are more exposed to an aging pig population and should keep neoplastic process in their differential.

One pig with histologic evidence of cerebral infarcts exhibited lesions with similar MRI features as cerebral infarcts reported in dogs (10, 11). Histopathologic findings were consistent with areas of necrosis corresponding to areas of presumptive infarcts on MRI. The infarcts were suspected to be secondary to mitral and aortic valve endocarditis. This case is a reminder that vascular and embolic events should be a differential diagnosis in pigs presented with neurological signs particularly in the presence of systemic comorbidities that could be associated with a hypercoagulable state or bacteremia which could impact the treatment and significantly alter the prognosis of these animals.

Another interesting case is the pig with the 4th ventricle diverticulum and associated obstructive hydrocephalus. Although an inflammatory process could not be completely excluded, a congenital cause was considered more likely for these findings. Similar findings have been reported in dogs (12, 13). Our case was treated with placement of a ventriculoperitoneal shunt allowing stabilization of its neurological signs, a procedure only reported in research pigs. Unfortunately, the pig was euthanized due to a suspected shunt infection, a relatively common complication with this procedure (14–16). Congenital anomalies should be included in the list of differential diagnoses in young pigs even with progressive signs. Although the CSF analysis showed a moderate pleocytosis in this animal, it was suspected that this change was secondary to the hydrocephalus and syringohydromyelia as reported in humans and dogs (17–19). More cases are needed to determine the prognosis for pigs with hydrocephalus treated with ventriculoperitoneal shunting, but clinicians should know that the procedure is feasible, similar to dogs, if owners are willing to try invasive treatment.

In one case, MRI identified muscle changes that were later attributed to an infection with *Erysipelothrix rhusiopathiae*. Erysipelas infection has been reported to cause fever as well as difficulty rising and walking secondary to pain and arthritis in pigs (1, 20). Other common causes of muscle disease in this species include metabolic causes such as vitamin E and/or selenium deficiency, trauma, bacterial infections (*Actinobacillus suis, Clostridium* sp.), Porcine Stress syndrome, Gossypol or Monensin toxicities, or parasitic myopathies (*Trichinella, Taenia*) (1). These diagnoses require histopathological evaluation of either a biopsy sample or the carcass post-mortem. Although MRI findings can help identify muscle involvement and can guide muscle sampling, it is unclear whether this imaging modality can help differentiate the different causes of myopathies in pigs.

Fibrocartilagenous embolisms (FCE) have been reported in several species including dogs, cats, sheep, and pigs (21–26). The mechanism for development of FCE is thought to be the result of a break-off of the nucleus pulposus entering the arteriolar system, commonly seen after vigorous exercise (27). In swine, FCE has been reported in 45 days old pigs (26), grow-finisher pigs (28), and a mature sow (25). In dogs, common causes of acute myelopathy include ANNPE and FCE, which can both present with similar neurologic signs of acute, non-progressive, lateralizing, and non-painful neurologic deficits (21, 29). Although a definitive diagnosis requires spinal cord histopathology, certain MRI changes can be identified to aid in an ante-mortem diagnosis of these conditions. FCE and ANNPE share similar MRI features including focal intramedullary T2W hyperintensity compared to normal spinal cord (21, 29, 30). Imaging features more consistent with ANNPE include lesion location centered over an intervertebral disc space, diminished T2W signal within the nucleus pulposus, and a narrowed intervertebral disc space, with minimal or no spinal cord compression at the affected level of the spinal cord (29, 30). Although ANNPE has not been described in pigs, the pigs described as cases 5 and 6 shared some of the imaging features described in dogs with ANNPE, suggesting a similar process may have occurred in these two animals. The previously reported cases of ischemic myelopathy were necropsy findings. Both pigs in this study recovered, suggesting that, as with dogs, neurological improvement is possible with these conditions and therefore euthanasia of these animals is not warranted if their neurological signs are not too severe (if deep pain perception persists) and supportive care can be provided.

Four of the pigs in our study had a normal MRI examination. One had an inflammatory CSF analysis, supporting that a meningitis cannot be excluded even in the face of a normal MRI examination. CSF analysis is thus indicated as an adjunct to MRI in pigs as it is in dogs (19). One pig presented with acute onset of vestibular signs, and no underlying cause for these signs was identified despite MRI, CSF analysis and infectious disease testing. Therefore, idiopathic vestibular syndrome was suspected. Vestibular signs in pigs are more commonly seen secondary to otitis media/interna and bacterial meningitis from *Streptococcus suis* infection (31). Idiopathic vestibular syndrome has been reported in dogs and cats (32–34) and remains a diagnosis of exclusion. To our knowledge, however, it has not yet been reported in pigs and therefore, this would be the first report of this diagnosis in this species. This case is interesting as MRI allowed to exclude intracranial causes of vestibular signs and prevented the animal from being euthanized just based on the severity of the neurological deficits. The recovery process took several months, but eventually the animal made a full recovery and the owners were pleased with the outcome.

Some limitations of the study are due to its retrospective nature. The medical records were incomplete in some cases, and the neurological examination not always fully reported. Additionally, not all cases were evaluated by a board certified neurologist, so some of the neurological deficits reported might not be fully accurate. Furthermore, details on onset of signs and progression were lacking for several cases. Finally, because only a few cases had histopathological diagnosis, several of our diagnosis are only presumptive.

As pot-bellied pigs become more popular pets, clinicians will likely encounter more neurological cases. This study shows that MRI can offer valuable ante-mortem information and provide clinicians with an important diagnostic tool to institute proper treatment and provide a prognosis of neurological conditions.

## Data Availability Statement

All datasets generated for this study are included in the article/Supplementary Material.

## Ethics Statement

Ethical review and approval was not required for the animal study because this is a retrospective study using data previously collected on client-owned animals treated at our institution with their owners' consent. This study did not require recruitment of any animal for the research purpose and no procedure was performed for the sole purpose of this project. Written informed consent was obtained from the owners for the participation of their animals in this study.

## Author Contributions

MV contributed to drafting the manuscript in collaboration with AC, who also contributed to article conception and design. AC, VD, and SH contributed to acquisition of the data (case recruitments, calling owners for update, and reviewing medical records). The MRI images were reviewed and interpreted by AC and SH. VD and SH participated in the revision of the article for its intellectual content. All authors contributed to the manuscript final revision and have read and approved the submitted manuscript.

### Conflict of Interest

The authors declare that the research was conducted in the absence of any commercial or financial relationships that could be construed as a potential conflict of interest.

## Abbreviations

ANNPE: Acute non-compressive nucleus pulposus extrusion
C: cervical
CBC: complete blood count
CSF: cerebrospinal fluid
FCE: fibrocartilaginous embolism
FLAIR: fluid attenuation inversion recovery
HASTE: half-Fourier acquisition single-shot turbo spin-echo
L: lumbar
MRI: Magnetic resonance imaging
T: thoracic
T1W: T1-weighed
T2W: T2-weighed
T2^*^GRE: T2^*^gradient recall echo
S: sacral
STIR: Short tau inversion recovery.
