# Supplementary material for: Magnetic Resonance Imaging Findings in 13 Neurologic Pot-Bellied Pigs
Source: Front Vet Sci. 2020 Jan 31;7:21. doi: 10.3389/fvets.2020.00021 (PMC7006440; doi:10.3389/fvets.2020.00021)
Supplement: Supplementary file 1 [file Table_1.docx]

**Table 1:** Signalment, duration (from onset to presentation at the referral hospital), onset and progression of clinical signs, neuroanatomical localization, cerebrospinal fluid (CSF) results (when applicable), histopathological results (when applicable), final diagnoses and follow up information in 13 pot-bellied pigs presented with neurological signs and that underwent magnetic resonance imaging (MRI). Results of the MRI findings are detailed in the text. Cases 10 to 13 are highlighted in grey, as their MRI did not reveal any structural lesion.

|  | **Age** | **Weight** | **Sex** | **Duration of signs** | **Onset and progression** | **Neurolocalization** | **CSF findings** | **Final Diagnoses** | **Follow up** |
| --- | --- | --- | --- | --- | --- | --- | --- | --- | --- |
| **1** | 18 mo | 45kg | MC | 10 days | Acute onset then waxing and waning signs | Presumptive L4-S3 myelopathy | Not performed | Myositis from *Erysipelothrix rhusiopathiae* | 5 days, improved |
| **2** | 13 yrs | 29.5kg | MC | 4 wks | Acute and non-progressive | T3-L3 myelopathy | Not performed | Round cell neoplasia | Euthanized |
| **3** | 6 mo | 28kg | MC | 4 days | Acute, non-progressive | T3-L3 myelopathy | Not performed | Presumptive myelomalacia | Euthanized |
| **4** | 10 yrs | 62.7kg | MC | 1 wk | Unclear onset, progressive | Brainstem or C1-C5 myelopathy | Not performed | Extradural cystic mass of unknown etiology | Euthanized |
| **5** | 1 yr | 55kg | FI | 1 mo | Acute, improving | T3-L3 myelopathy | Not performed | Presumptive FCE or ANNPE | LTFU |
| **6** | 9 mo | 50kg | FI | <24h | Acute, static | L4-S3 myelopathy | Not performed | Presumptive ANNPE | 2 yrs, improved (occasional ataxia) |
| **7** | 3 mo | 10.6 | FI | 10 days | Unclear | L4-S3 myelopathy | Not performed | 1^st^ sacral vertebrae fracture with sacral nerve root laceration | 3 weeks, doing well after amputation |
| **8** | 7 yrs | 65kg | MC | 2 wks | Progressive onset, worsening signs | Forebrain or brainstem | Not performed | Moderate multifocal acute and chronic encephalomalacia with thrombi (infarcts) | Euthanized |
| **9** | 10 mo | 15kg | MC | 2 wks | Insidious onset, Progressive | Central vestibular system | 1. Mixed, predominantly mononuclear pleocytosis  2. Neutrophilic pleocytosis | Obstructive hydrocephalus secondary to fourth ventricle dilation | Euthanized 2 mo after VP shunt placement due to suspected infection. |
| **10** | 5 mo | 14kg | MC | 24h | Acute, static | Vestibular system | Mixed (primarily mononuclear) pleocytosis | Meningoencephalitis | LTFU |
| **11** | 6 yr | 33kg | FS | 19h | Acute, improving | Vestibular system | Normal | Idiopathic vestibular | Improved over 5 months |
| **12** | 3 yr | 145kg | MC | 7h | Acute, improving | Forebrain | N/A | Unknown | 3 yrs, no seizure. |
| **13** | 3 mo | 7.2kg | MI | 1 wk | Unclear | L4-S3 myelopathy | N/A | Unknown | Improved 3 months later |

ANNPE: Acute non-compressive nucleus pulposus extrusion, C: cervical, CSF: Cerebrospinal fluid, FCE: Fibrocartilaginous embolism, FI: female intact, FS: Female spayed, h: hours, L: lumbar, LTFU: Lost to follow up, MC: Male castrated, MI: Male intact, mo, months, N/A: not applicable, NR: not recorded, S: sacral, T: thoracic, VP: Ventriculoperitoneal, wk: week, yr: year.
